# YOLO-Shrimp: A Lightweight Detection Model for Shrimp Feed Residues Fusing Multi-Attention Features

**DOI:** 10.3390/s26030791

**Published:** 2026-01-24

**Authors:** Tianwen Hou, Xinying Miao, Zhenghan Wang, Yi Zhang, Zhipeng He, Yifei Sun, Wei Wang, Ping Ren

**Affiliations:** 1 College of Information Engineering, Dalian Ocean University, Dalian 116023, China; mxylw@dlou.edu.cn (T.H.); 19883296810@163.com (Z.W.); 15175596813@163.com (Y.Z.); 15002400885@163.com (Z.H.); 13841725679@163.com (Y.S.); ww_wangwei@dlou.edu.cn (W.W.); renping@dlou.edu.cn (P.R.); 2 Liaoning Provincial Key Laboratory Marine Informat Technology, Dalian 116023, China

**Keywords:** shrimp farming, aquaculture, YOLO, deep learning

## Abstract

Precise control of feeding rates is critically important in intensive shrimp farming for cost reduction, optimization of farming strategies, and protection of the aquatic environment. However, current assessment of residual feed in feeding trays relies predominantly on manual visual inspection, which is inefficient, highly subjective, and difficult to standardize. The residual feed particles typically exhibit characteristics such as small size, high density, irregular shapes, and mutual occlusion, posing significant challenges for automated visual detection. To address these issues, this study proposes a lightweight detection model named YOLO-Shrimp. To enhance the network’s capability in extracting features from small and dense targets, a novel attention mechanism termed EnSimAM is designed. Building upon the SimAM structure, EnSimAM incorporates local variance and edge response to achieve multi-scale feature perception. Furthermore, to improve localization accuracy for small objects, an enhanced weighted intersection over union loss function, EnWIoU, is introduced. Additionally, the lightweight RepGhost module is adopted as the backbone of the model, significantly reducing both the number of parameters and computational complexity while maintaining detection accuracy. Evaluated on a real-world aquaculture dataset containing 3461 images, YOLO-Shrimp achieves mAP@0.5 and mAP@0.5:0.95 scores of 70.01% and 28.01%, respectively, while reducing the parameter count by 19.7% and GFLOPs by 14.6% compared to the baseline model.

## 1. Introduction

Shrimp farming constitutes a vital component of global aquaculture, with its industrial scale and economic value continuing to grow. In practice, feed costs typically account for over 50% of total production expenses, making them a critical factor influencing farming profitability [1]. Overfeeding not only leads to direct feed wastage and increased operational costs, but uneaten residual feed decomposes in water, releasing harmful substances such as ammonia and nitrite, thereby deteriorating water quality and potentially triggering shrimp diseases that threaten the stability of the aquaculture ecosystem [2]. Conversely, underfeeding impedes normal shrimp growth, reducing both yield and market size [3]. Therefore, achieving rapid and accurate assessment of residual feed in feeding trays after feeding is essential for optimizing feeding strategies, reducing costs, improving efficiency, and promoting sustainable aquaculture practices.

Currently, monitoring of residual feed in aquaculture relies heavily on empirical visual inspection by farm workers, who subjectively evaluate feed consumption in trays to adjust feeding amounts [4]. This traditional approach suffers from several limitations: it is labor-intensive and inefficient, unsuitable for large-scale, high-density farming operations [5,6,7]; its outcomes depend strongly on individual experience, lacking objective and consistent standards, which leads to arbitrary and unstable feeding decisions; and manual inspections cannot provide continuous, high-frequency monitoring, potentially missing optimal adjustment opportunities [8,9]. Collectively, these issues hinder the advancement of precision and intelligent shrimp farming.

**Figure 1:**
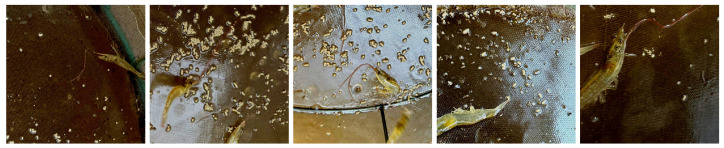
Presentation of partial images in real-world tasks.

With the rapid development of computer vision and deep learning, image-based automated monitoring in aquaculture has emerged as an active research field [10,11,12]. Deep learning-based object detection algorithms represented by the YOLO (You Only Look Once) series have been widely adopted in various aquaculture applications, such as fish counting, behavioral analysis and growth monitoring, owing to their favorable trade-off between inference speed and detection accuracy. However, the direct application of general-purpose detection models to the task of uneaten feed monitoring in shrimp farming is confronted with unique performance bottlenecks [13]. Specifically, existing methods exhibit several major failure modes in this task: (1) Severe missed detections occur in high-density uneaten feed regions, where the model struggles to distinguish densely clumped feed particles; (2) confusion with tray textures or reflective areas gives rise to false alarms; and (3) inaccurate localization is observed for slender or irregularly shaped particles. These deficiencies stem from the fact that general-purpose models are not optimized for handling targets characterized by extreme small size, dense distribution and morphological diversity. Their standard architectures are difficult to effectively extract subtle features in complex underwater environments (e.g., variable illumination and turbid water). As illustrated in Figure 1, the intricate background and color ambiguity render ordinary deep learning models incompetent for this task.

To address these challenges, this paper proposes an improved model named YOLO-Shrimp, which is based on the lightweight YOLOv11n architecture and optimized specifically for uneaten feed detection in shrimp farming. Our objective is to develop a lightweight solution that satisfies the requirements of low deployment cost and real-time processing capability while maintaining high detection accuracy. By resolving the aforementioned failure modes, YOLO-Shrimp equips aquaculture farm operators with concrete decision-making capabilities that are either unachievable or unreliable with existing manual inspection methods or benchmark detection models. For instance, through more accurate quantification of uneaten feed, the system enables automatic and precise adjustment of feed dosage, thereby avoiding feed waste and reducing production costs. Meanwhile, continuous high-frequency monitoring allows for earlier identification of abnormal feeding behaviors caused by diseases or abrupt environmental changes compared with manual observation, which gains valuable time for early warning and intervention. The main contributions of this work are summarized as follows:

- Model Architecture Innovation: Introduced a RepGhost-based lightweight backbone, reducing parameters by 19.7% and GFLOPs by 14.6% while improving mAP@0.5 by 2.80 percentage points, achieving dual enhancement of efficiency and accuracy.
- Attention Mechanism Design: Proposed EnSimAM, a parameter-free multi-scale attention mechanism fusing global, local and edge responses, improving mAP@0.5 by 1.27 percentage points without extra computation and enhancing dense small-target feature extraction.
- Loss Function and Optimization: Designed EnWIoU with small-target morphological/directional constraints, improving mAP@0.5 by 1.05 percentage points and enhancing irregular uneaten feed localization.
- Dataset and Application Validation: YOLO-Shrimp validated on 3461-image real farm dataset, achieving 70.01% mAP@0.5 and 28.01% mAP@0.5:0.95 (2.08 M params, 5.5 GFLOPs), outperforming advanced lightweight detectors and verifying practical performance.

## 2. Related Works

In recent years, deep learning-based object detection has witnessed substantial progress [14,15,16], with mainstream methods generally categorized into two-stage and single-stage detectors. Representative two-stage detectors, such as the R-CNN series [17], first generate region proposals and then perform classification and regression on these regions; while these approaches typically achieve high accuracy, they suffer from high computational complexity and often fail to meet real-time requirements. In contrast, single-stage detectors like YOLO (You Only Look Once) [18] and SSD (Single Shot MultiBox Detector) [19] formulate object detection as a regression problem, directly predicting bounding boxes and class probabilities from images. This paradigm effectively balances speed and accuracy, making such models highly suitable for real-world applications.

A significant challenge in object detection is the accurate localization of small objects, which often occupy only a few pixels and lack sufficient feature information [20]. This issue is particularly prevalent in applications such as industrial defect detection and autonomous driving. To address this, researchers have explored various strategies. Multi-scale feature fusion, as seen in Feature Pyramid Networks, has become a standard for enhancing semantic information across different scales. Data augmentation techniques, including random cropping, rotation, and advanced methods like Mosaic, are widely used to increase the diversity of small objects in training data [21]. Furthermore, some studies have employed Generative Adversarial Networks (GANs) to synthesize additional training samples, particularly for rare or densely distributed industrial defects, thereby improving model robustness.

The YOLO series stands out as a prominent representative of single-stage detectors. Through successive iterations from YOLOv1 to YOLOv4, the framework has been continuously optimized in terms of backbone architecture, feature fusion strategies, and loss functions [22]. YOLOv5 achieved notable engineering success by offering multiple model sizes tailored to diverse application scenarios [23]. Subsequent releases including YOLOv8 and YOLOv10 further introduced novel architectural designs and training strategies, consistently advancing the state-of-the-art in real-time object detection [24]. YOLOv9 [25] introduces two key innovations: Programmable Gradient Information (PGI) and the Generalized Efficient Layer Aggregation Network (GELAN). PGI mitigates the information bottleneck by using an auxiliary reversible branch to generate reliable gradients, ensuring that complete feature information is available for the loss function. GELAN, a lightweight and efficient architecture based on gradient path planning, combines the advantages of CSPNet and ELAN to achieve superior parameter utilization and faster inference speeds using only conventional convolution operators. The recent YOLOv11 iteration incorporates innovative modules such as C3k2, SPPF, and C2PSA, enhancing feature extraction, multi-scale fusion, and spatial attention mechanisms, thereby establishing a new benchmark for high-precision and efficient detection [26]. Nevertheless, even advanced YOLO models exhibit limitations when detecting extremely small and dense objects like shrimp feed residues, highlighting the need for specialized optimizations for small object detection [27].

To enable the deployment of deep learning models on resource-constrained edge devices, lightweight network design has emerged as a critical research direction. The MobileNet [28] and ShuffleNet [29] series effectively reduce parameter counts and computational costs through techniques including depthwise separable convolutions, group convolutions, and channel shuffling [30]. GhostNet further minimizes redundancy by generating “ghost” feature maps with minimal computation. Building on these advances, RepGhost incorporates re-parameterization technology, utilizing multi-branch structures during training to enhance representational capacity while equivalently converting to a single-path architecture during inference [31]. This “training-inference decoupling” paradigm provides a promising direction for developing efficient yet powerful lightweight models.

The integration of attention mechanisms has significantly enhanced the feature representation capabilities of neural networks by enabling dynamic focus on informative regions of input data. The SE module recalibrates channel-wise feature responses by learning inter-channel importance, while CBAM combines both channel and spatial attention. However, most existing approaches introduce additional parameters [32]. SimAM presents a parameter-free 3D attention mechanism grounded in neuroscience theory, defining an energy function to compute the importance of each neuron and enabling refined weight allocation across feature maps [33]. Despite its simplicity and effectiveness, SimAM’s global perception characteristics may inadequately capture locally critical information when handling challenging small targets, leaving room for targeted improvements in attention design for such scenarios.

Loss functions play a pivotal role in guiding model optimization for object detection; while the traditional IoU loss is intuitive, it suffers from gradient vanishing when predicted and ground-truth boxes exhibit no overlap. To address this limitation, various improved IoU-based losses have been proposed [34]. GIoU introduces a minimum enclosing box to penalize non-overlapping cases, while DIoU directly incorporates the normalized distance between box centers to accelerate convergence [35]. CIoU further extends DIoU by considering aspect ratio consistency. However, these loss functions can be dominated by large gradients from low-quality samples when handling datasets with varying annotation quality [36]. WIoU addresses this issue through a dynamic non-monotonic focusing mechanism that intelligently assigns gradient gains by evaluating the “outlier degree” of anchor boxes. By reducing the weights of both high-quality and low-quality samples, WIoU enables the model to focus more effectively on learning from “ordinary” quality samples, thereby enhancing overall generalization capability [37,38]. This conceptual framework provides valuable inspiration for optimizing small object localization.

Computer vision technologies have demonstrated significant potential in intelligent aquaculture applications. Researchers have utilized these techniques for fish counting, size measurement, behavior analysis, and disease diagnosis [39]. Particularly in feeding management, visual methods for monitoring feeding behavior and residual feed levels have gained increasing research attention [40]. For instance, several studies have analyzed fish movement patterns and aggregation density to assess feeding intensity. For residual feed detection, some earlier works employed traditional image processing methods, though these approaches often lack robustness in complex underwater environments [41]. More recently, deep learning-based methods have been applied to residual feed identification and counting, including density estimation networks for statistically quantifying densely distributed residues [42,43]. Despite these preliminary explorations, there remains a notable research gap in developing efficient, lightweight detection models specifically designed for identifying small and dense residual feed in shrimp feeding trays. Motivated by this challenge, our study integrates recent advances in object detection, lightweight networks, and attention mechanisms to provide an innovative solution to this pressing industry problem.

## 3. Proposed Methods

**Figure 2:**
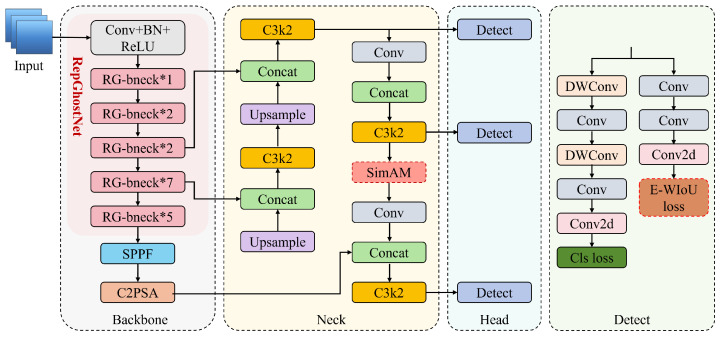
Overall Architecture of YOLO-Shrimp, where dashed lines denote our improved modules, red backgrounds represent the entirely optimized components, and the symbol ‘*’ denotes the number of repetitions of the corresponding module.

To address the challenges of detecting small, dense shrimp feed residues in complex aquatic environments, we have introduced a series of targeted improvements to the YOLOv11n architecture, resulting in a lightweight detection framework named YOLO-Shrimp that balances high accuracy with computational efficiency. The overall architecture of our proposed model is illustrated in Figure 2.

The end-to-end process of YOLO-Shrimp is as follows: an input image is first processed by the lightweight RepGhost backbone network to extract multi-scale feature maps. These features are then fed into the neck network, where our proposed EnSimAM attention modules are strategically placed to enhance the features of small targets. The fused feature maps are subsequently passed to the detection head, which generates the final predictions for bounding boxes and classes. During the training phase, the discrepancy between the predicted and ground-truth boxes is measured by our custom EnWIoU loss function, which provides precise gradient information to guide model optimization. While our core components, EnSimAM and EnWIoU, are integrated into the YOLO framework, they represent significant conceptual departures from existing methods. EnSimAM distinguishes itself from SimAM and other attention mechanisms through its unique three-branch parallel design, which simultaneously models global neuronal importance, local feature variance, and edge information. Unlike SimAM, which relies solely on a global energy function, or other mechanisms that focus on channel and spatial dimensions, EnSimAM’s composite structure is specifically engineered to capture the multifaceted characteristics of small, irregular objects. Similarly, EnWIoU is not a mere incremental improvement upon IoU-based losses like CIoU, DIoU, or WIoU. It introduces highly specific geometric constraints—namely, orientation consistency and aspect ratio sensitivity—that are tailored to the unique morphology of shrimp feed residues. These are not general-purpose extensions but targeted modifications designed to provide more accurate localization supervision for the small, elongated objects that are central to our detection task, a level of specificity not found in its predecessors.

### 3.1. EnSimAM

**Figure 3:**
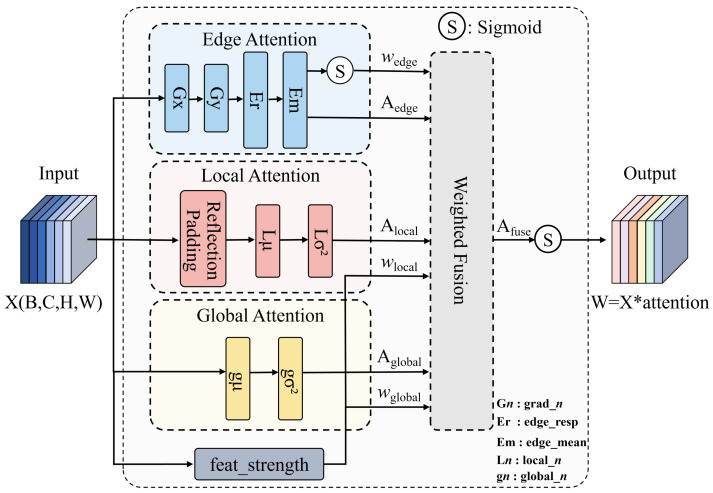
Overall Structure Diagram of EnSimAM.

Standard attention mechanisms exhibit excellent performance in processing large-scale features; however, their global receptive fields often fail to capture critical local details of targets with minimal pixel coverage. Inspired by the parameter-free design paradigm of SimAM, we propose an enhanced multi-scale attention mechanism termed EnSimAM, whose architectural details are illustrated in Figure 3. Specifically tailored for dense small-object detection scenarios, this mechanism integrates global contextual information, local fine-grained details, and edge semantic cues to construct a more comprehensive and robust feature representation. The core design logic of EnSimAM resides in the parallel computation and adaptive fusion of three distinct dimensional attention cues.

Specifically, given an input feature map $X\in R^{C\times H\times W}$, where *C*, *H*, and *W* denote the number of channels, height, and width, respectively, we construct three parallel attention branches: Global Attention, Local Variance Enhancement, and Edge Response Enhancement. In the Global Attention branch, we preserve the core concept of the original SimAM mechanism by defining an energy function to evaluate the importance of each neuron. For a target neuron *t* and other neurons *i* in the feature map, the energy function is defined as:

(1) $$e_{t}\left(w_{t},b_{t},y,x_{i}\right)=\frac{1}{M-1}\sum_{i=1}^{M-1}{\left(1-\left(w_{t}x_{i}+b_{t}\right)\right)}^{2}+\frac{1}{2}\left(w_{t}^{2}+b_{t}^{2}\right)$$

Specifically, where M=H×W represents the total number of neurons in the channel, and $w_{t}$ and $b_{t}$ denote the weight and bias of the linear transformation, respectively. By minimizing this energy function, we derive the closed-form solution as follows:

(2) $$e_{t}^{*}=\frac{4({\hat{\sigma }}^{2}+\lambda )}{{\left(t-\hat{\mu }\right)}^{2}+2{\hat{\sigma }}^{2}+2\lambda }$$

Specifically, where $\hat{\mu }$ and ${\hat{\sigma }}^{2}$ represent the mean and variance of the channel features, respectively, and λ denotes the regularization coefficient. The global attention weights are then computed as:

(3) $$A_{global}=\mathrm{sigmoid}\left(\frac{1}{e_{t}^{*}}\right)$$

To enhance the feature variations of small targets within local regions, we perform local variance enhancement by first obtaining the local mean $\mu_{local}$ through 3 × 3 average pooling, followed by computing the local variance as:

(4) $$\begin{array}{c} \mu_{\mathrm{local}\;}\left(x,y\right)=\frac{1}{9}\sum_{i=-1}^{1}\sum_{j=-1}^{1}X\left(x+i,y+j\right)\\ \sigma_{\mathrm{local}\;}^{2}\left(x,y\right)=\frac{1}{9}\sum_{i=-1}^{1}\sum_{j=-1}^{1}{\left(X\left(x+i,y+j\right)-\mu_{\mathrm{local}\;}\left(x,y\right)\right)}^{2} \end{array}$$

Local attention weights are defined as:

(5) $$A_{local}=\mathrm{sigmoid}\left(\alpha \cdot \sigma_{local}^{2}\right)$$

where α denotes a scaling factor that regulates the response intensity of the local variance. In the corresponding Edge Response Enhancement branch, we compute the horizontal and vertical gradients using an approximated Sobel operator. The horizontal and vertical gradient components are, respectively, defined as:

(6) $$\begin{array}{c} G_{x}=\left[\begin{array}{ccccccc} -1 & 0 & 1-2 & 0 & 2-1 & 0 & 1 \end{array}\right]*X\\ G_{y}=\left[\begin{array}{ccccccc} -1 & -2 & -10 & 0 & 01 & 2 & 1 \end{array}\right]*X \end{array}$$

The edge strength is computed as:

(7) $$G_{edge}=\sqrt{G_{x}^{2}+G_{y}^{2}}$$

Subsequently, the edge attention weights are derived as:

(8) $$A_{edge}=\mathrm{sigmoid}\left(\beta \cdot G_{edge}\right)$$

where β serves as a scaling factor for the edge response intensity. These fusion weights are dynamically computed based on the energy of the input feature map itself and are not learned parameters, ensuring the mechanism remains parameter-free. The three types of attention are not simply added together, but fused through adaptive weights. We calculate the fusion weights based on feature intensity as follows:

(9) $$\omega_{global}=\frac{{\left|X\right|}^{2}}{{\left|X\right|}^{2}+\epsilon },\;\omega_{local}=\omega_{edge}=\frac{1-\omega_{global}}{2}$$

where ϵ is a small constant included to prevent division by zero. The final attention weights are then obtained as follows:

(10) $$A_{EnSimAM}=\omega_{global}\cdot A_{global}+\omega_{local}\cdot A_{local}+\omega_{edge}\cdot A_{edge}$$

### 3.2. EnWIoU

The precision of bounding box regression is particularly critical for small object detection; while WIoU demonstrates remarkable performance through its dynamic focusing mechanism when handling training samples of varying quality, scenarios such as residual feed detection present additional challenges—targets not only exhibit small sizes but also frequently assume irregular elongated shapes. To further enhance localization accuracy, we propose an Enhanced WIoU loss function (EnWIoU). In the foundational WIoU formulation, given a predicted box $B^{p}=\left(x^{p},y^{p},w^{p},h^{p}\right)$ and a ground truth box $B^{gt}=\left(x^{gt},y^{gt},w^{gt},h^{gt}\right)$, where (x,y) denotes the center coordinates and *w* and *h* represent the width and height, respectively, the IoU is defined as:

(11) $$\mathrm{IoU}=\frac{|B^{p}\cap B^{gt}|}{|B^{p}\cup B^{gt}|}$$

WIoU introduces a dynamic non-monotonic focusing mechanism that adaptively adjusts the loss weight $L_{WIoU}=r\cdot L_{IoU}$ by evaluating the outlier degree β of anchor boxes. Here, $r=\beta^{\delta \alpha \beta^{-\alpha }}$ represents an intelligent gradient gain allocation strategy, with α and δ being hyperparameters. The outlier degree β is defined as:

(12) $$\beta =\frac{d}{C}=\frac{\sqrt{{\left(x^{gt}-x^{p}\right)}^{2}+{\left(y^{gt}-y^{p}\right)}^{2}}}{\sqrt{w_{g}^{2}+h_{g}^{2}}}$$

where *d* denotes the Euclidean distance between the centers of the predicted and ground-truth bounding boxes, and *C* represents the diagonal length of their minimum enclosing bounding box. Building upon WIoU, we introduce two geometric constraint factors specifically designed for small object characteristics. First, we compute the aspect ratios of both predicted and ground-truth boxes as follows:

(13) $$r_{aspect}^{p}=\frac{\max(w^{p},h^{p})}{\min(w^{p},h^{p})},\;r_{aspect}^{gt}=\frac{\max(w^{gt},h^{gt})}{\min(w^{gt},h^{gt})}$$

When $\max\left(r_{aspect}^{p},r_{aspect}^{gt}\right)>\tau_{aspect}$ (where $\tau_{aspect}=2.0$ serves as the threshold), the target is identified as an elongated small object. Subsequently, we define a directional consistency indicator function as follows:

(14) $$I_{\mathrm{orient}\;}=\{\begin{array}{cc} 1, & \;\mathrm{if}\;\left(w^{p}>h^{p}\right)\wedge \left(w^{gt}>h^{gt}\right)\;\mathrm{or}\;\left(w^{p}\leq h^{p}\right)\wedge \left(w^{gt}\leq h^{gt}\right)\\ 0, & \;\mathrm{otherwise}\; \end{array}$$

**Figure 4:**
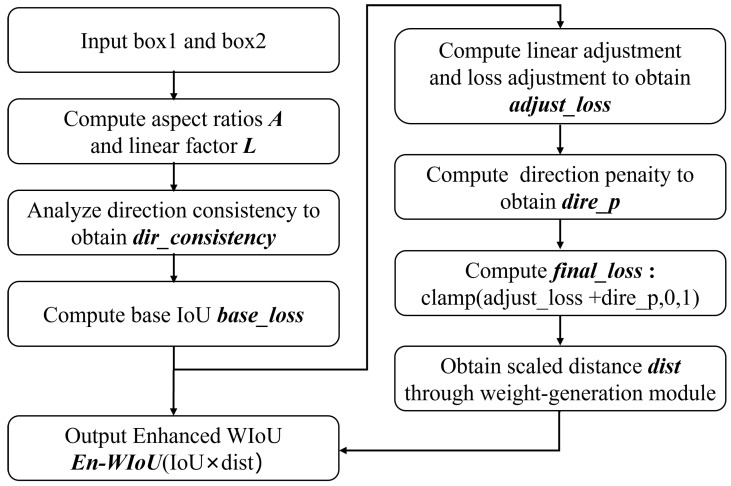
EnWIoU Loss Function Flow Chart.

This indicator function determines whether the predicted bounding box and the ground truth bounding box are consistent in their main directions (both horizontal or both vertical). Incorporating the aforementioned two constraints, we define the EnWIoU loss as follows:

(15) LEnWIoU=clamp(r·LIoU·γ,0,1)

where the orientation modulation factor γ is defined as:

$$\gamma =\left\{\begin{array}{cc} 1-\eta \cdot I_{\mathrm{orient}\;}, & \;\mathrm{if}\;\max\left(r_{\mathrm{aspect}\;}^{p},r_{\mathrm{aspect}\;}^{gt}\right)>\tau_{\mathrm{aspect}\;}\\ 1+\eta \cdot \left(1-I_{\mathrm{orient}\;}\right), & \;\mathrm{if}\;\max\left(r_{\mathrm{aspect}\;}^{p},r_{\mathrm{aspect}\;}^{gt}\right)>\tau_{\mathrm{aspect}\;}\\ 1, & \;\mathrm{otherwise}\; \end{array}\right.$$

where η denotes the orientation penalty intensity coefficient, which is set to 0.1 in our experiments. When an elongated object is detected with consistent orientation, γ<1 reduces the loss weight; conversely, when orientation inconsistency occurs, γ>1 increases the penalty. Finally, a clamp function is applied to confine the loss value within the range [0, 1], ensuring training stability. As illustrated in Figure 4, this design enables EnWIoU to not only inherit WIoU’s intelligent sensitivity to sample quality but also incorporate refined considerations for target morphology and orientation. Consequently, it achieves more robust and precise supervision in localization tasks involving small targets, particularly those with irregular shapes.

### 3.3. RepGhost

**Figure 5:**
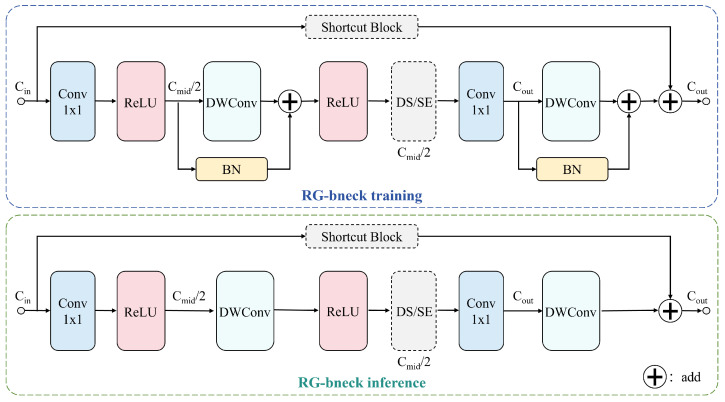
Structure Diagram of the RepGhost Lightweight Backbone.

To develop a lightweight detection model suitable for edge computing applications, we have optimized the backbone network of YOLOv11n by incorporating RepGhost modules. RepGhost represents an efficient re-parameterization block that employs distinct architectures during training and inference phases to achieve “lossless” performance acceleration, with its detailed structure illustrated in Figure 5. The design of RepGhost modules draws inspiration from GhostNet. Given an input feature map $X\in R^{c\times h\times w}$, a conventional convolutional layer requires *n* convolutional kernels to generate *n* output feature maps. In RepGhost, we first utilize *m* (m<n) convolutional kernels to produce intrinsic features $Y^{\prime }$ as follows:

(16) $$Y^{\prime }=X*f^{\prime },\;Y^{\prime }\in R^{m\times h\times w}$$

where $f^{\prime }$ denotes the primary convolution operation. Subsequently, a series of inexpensive linear transformations Φ are applied to each intrinsic feature to generate ghost features according to $y_{ij}=\Phi_{i,j}\left(y_{i}^{\prime }\right),\;\forall i=1,\ldots ,m,\;j=1,\ldots ,s$, where $s=\frac{n}{m}$ represents the number of ghost features produced from each intrinsic feature. The final output is obtained by concatenating all generated features as follows:

(17) $$Y=\left[Y^{\prime },Y^{\prime \prime }\right],\;Y\in R^{n\times h\times w}$$

This approach effectively reduces the computational complexity from $O(n\cdot c\cdot k^{2}\cdot h\cdot w)$ to $O(m\cdot c\cdot k^{2}\cdot h\cdot w+\left(s-1\right)\cdot m\cdot d^{2}\cdot h\cdot w)$, where *k* and *d* represent the kernel sizes of the primary convolution and linear operations, respectively.

### 3.4. Overall Algorithm of YOLO-Shrimp

To provide a clear overview of the model’s operational flow, the overall training and inference algorithm for YOLO-Shrimp is summarized in Table 1. The algorithm details how the input image is processed through the network and how the custom modules—RepGhost, EnSimAM, and EnWIoU—are integrated and interact within the YOLOv11n framework.

## 4. Experiment and Result Analysis

### 4.1. Dataset and Environment

This study utilizes a dataset collected from real-world shrimp farming environments. At a shrimp farming base in Xingshutun, Dalian, we captured images of feeding trays using conventional smartphones one hour after feeding. This specific timing was selected because partial feed consumption has typically occurred by this point, making the distribution and morphology of residual feed most representative for assessment purposes. The original dataset comprised 1200 images. Given the small size and morphological uniformity of residual feed particles, we performed data preprocessing and augmentation operations to expand the dataset scale, enhance sample diversity, and prevent model overfitting. These operations included random cropping, rotation, flipping, and brightness/contrast adjustments. After processing, the final dataset was expanded to 3461 images. These images authentically reflect the challenges inherent in residual feed detection, including dense distribution of fine particles, size variation, mutual adhesion, and uneven underwater illumination. The entire dataset was randomly split into training and validation sets following a 9:1 ratio, resulting in 3115 images for training and 346 images for validation. All experiments were conducted under consistent hardware and software configurations to ensure the reproducibility of results. The detailed experimental environment specifications are summarized in Table 2.

To quantitatively evaluate model performance, we adopted widely recognized metrics in object detection, including Precision (P), Recall (R), mean Average Precision (mAP), number of parameters, and Giga Floating Point Operations (GFLOPs).

### 4.2. Comparative and Ablation Experiments

To further demonstrate the superiority of our proposed model, we conducted comprehensive comparisons with various state-of-the-art object detection algorithms, including other lightweight YOLO series models (YOLOv5n, YOLOv8n, YOLOv10n), classical detectors (Faster R-CNN, SSD), and more recent architectures (RT-DETR). All models were trained and evaluated on our dataset using identical experimental settings, with the comparative results summarized in Table 3.

Analysis of the experimental results reveals that our model substantially outperforms classical detectors such as SSD and Faster R-CNN across all accuracy metrics, while simultaneously maintaining significantly smaller model size and computational requirements. This demonstrates the inherent advantages of the YOLO architecture for real-time detection tasks. Compared with RT-DETR, another real-time oriented detector, our model achieves a 27.61 percentage point higher mAP@0.5 while utilizing only 10.9% of the parameters and 10.2% of the computational load, indicating a decisive advantage. In comparisons with lightweight models from the YOLO family, our approach consistently delivers the best performance. It surpasses YOLOv5n, YOLOv8n, and YOLOv10n by at least 5.44 percentage points in mAP@0.5 while achieving the lowest parameter count and GFLOPs among all compared YOLO variants. These results strongly validate the effectiveness of our improvement strategies. Notably, compared with the baseline YOLOv11n, our model not only achieves comprehensive accuracy improvements but also succeeds in reducing both model size and computational cost. This demonstrates that our method effectively optimizes and advances state-of-the-art performance for the specific and challenging task of residual feed detection, achieving an optimal balance among accuracy, speed, and model complexity.

To validate the effectiveness of each proposed improvement module, we conducted a series of ablation studies using YOLOv11n as the baseline model. The experimental results presented in Table 4 lead to the following conclusions: The original YOLOv11n model achieved 65.88% mAP@0.5 on our dataset, establishing a solid baseline for subsequent improvements. When integrating RepGhost as the backbone network, the model’s mAP@0.5 increased by 2.80 percentage points to 68.68%, while mAP@0.95 improved by 2.31 percentage points. Simultaneously, the parameter count and GFLOPs were significantly reduced by 19.7% and 14.6%, respectively. This demonstrates that the RepGhost module effectively enhances feature extraction capability while reducing model complexity, achieving dual improvements in both accuracy and efficiency. The individual incorporation of the EnSimAM attention mechanism into the baseline model resulted in a 1.27 percentage point gain in mAP@0.5. This indicates that our proposed multi-scale attention mechanism enables the model to more effectively focus on small targets like residual feed, thereby improving detection accuracy without introducing additional parameters. When exclusively employing EnWIoU as the loss function during training, the model achieved a 1.05 percentage point improvement in mAP@0.5. This verifies that the geometric constraints specifically designed for small target morphology and orientation provide more precise supervision for bounding box regression, consequently enhancing localization quality. The complete integration of all three modules yielded optimal performance, with mAP@0.5 and mAP@0.95 reaching 70.01% and 28.01%, respectively. These represent improvements of 4.13 and 2.93 percentage points over the baseline model. Both precision and recall rates also showed comprehensive enhancement. These results demonstrate that our three proposed enhancement modules exhibit strong synergistic effects, operating collaboratively across different aspects of the model to ultimately achieve state-of-the-art detection performance. To quantitatively assess the contribution of our data augmentation strategies, we conducted a series of ablation experiments. We started with a baseline model trained without any data augmentation and progressively introduced our complete and local enhancement strategies. The “complete data augmentation” includes standard geometric and photometric transformations, while the “local data augmentation” refers to targeted enhancements for small, dense objects, such as random cropping and pasting of residual feed particles. The results are detailed in Table 5. The results clearly demonstrate the effectiveness of data augmentation. The model trained without any augmentation achieved a baseline mAP@0.5 of 65.32%. Introducing the complete augmentation strategy provided a significant boost of 2.83 percentage points. Notably, the local augmentation strategy, which is specifically designed to address the challenges of small and dense objects, proved to be even more effective, yielding a 3.92 percentage point increase over the baseline. The best performance was achieved when both complete and local augmentation strategies were used in conjunction, resulting in a final mAP@0.5 of 70.01%, a 4.69 percentage point improvement over the non-augmented baseline. This confirms that a combination of general and targeted augmentation is crucial for achieving robust detection of small-sized, high-density residual feed particles.

To further clarify the sources of performance differences between YOLO-Shrimp and YOLOv9, we conducted a more in-depth quantitative analysis of the contributions of each core optimization module in the model. Although YOLO-Shrimp demonstrates significant advantages over YOLOv9 in macro indicators, the root cause of its performance improvement lies in the intricate synergistic effect among our proposed RepGhost backbone, EnSimAM attention mechanism, and EnWIoU loss function—with the RepGhost backbone being the primary driving force behind the performance gains. Integrating the RepGhost module alone yields a 2.80 percentage point increase in mAP@0.5, accounting for approximately 68% of the total performance improvement compared to the baseline model. More importantly, while enhancing accuracy, this module reduces the model parameter count from 2.59M to 2.08M and computational complexity from 6.44G to 5.50G. This characteristic directly explains why YOLO-Shrimp can achieve higher detection accuracy while having a lower computational cost than YOLOv9, establishing its core advantages in lightweight design and efficiency. The EnSimAM attention mechanism and EnWIoU loss function provide critical accuracy optimizations. As a plug-and-play module without additional parameters, EnSimAM contributes a 1.27 percentage point increase in mAP@0.5, demonstrating its effectiveness in enhancing feature perception capability for small targets without imposing extra model burden. Similarly, the EnWIoU loss function achieves a 1.05 percentage point gain in mAP@0.5 by optimizing sample weights and localization loss during training, with this improvement not affecting the final inference speed at all. Overall, it is the organic combination of these three modules—RepGhost significantly improving efficiency and basic performance, supplemented by EnSimAM and EnWIoU for refined tuning of features and loss—that collectively enables YOLO-Shrimp to outperform YOLOv9 with exceptional performance.

To further dissect the contributions of individual components within the EnSimAM and EnWIoU modules to model performance, we designed more fine-grained ablation experiments. As shown in Table 6, we first evaluated the three core components of EnSimAM—Global Attention, Local Attention, and Edge Attention—one by one. The experimental results indicate that integrating only SimAM-based Global Attention improved the model’s mAP@0.5 by 0.70 percentage points compared to the baseline, verifying its fundamental effectiveness in capturing global contextual information. Building on this, introducing Local Attention yielded an additional 0.30 percentage point gain, demonstrating the importance of focusing on local details in dense scenarios. Finally, combining Edge Attention (for strengthening edge responses) boosted the model’s performance to 67.15%, a further 0.27 percentage point improvement over the version using only Global and Local Attention. This clearly proves that EnSimAM achieves comprehensive enhanced perception of small objects by fusing feature cues from three distinct dimensions: Global Attention is the core driver of performance improvement, while Local and Edge Attention provide critical marginal gains. Next, we conducted a decoupled analysis of the two key designs of the EnWIoU loss function: Aspect Ratio Constraint and Directional Consistency Constraint. The baseline model uses standard WIoU loss; on this basis, introducing the Aspect Ratio Constraint alone improved mAP@0.5 by 0.42 percentage points, benefiting from its more accurate geometric modeling of elongated residual feed particles. Introducing the Directional Consistency Constraint alone brought a 0.25 percentage point performance improvement, indicating that supervision of target orientation helps enhance localization accuracy. When both constraints are enabled, EnWIoU increases mAP@0.5 to 66.93%—0.38 and 0.60 percentage points higher than the versions using only the Aspect Ratio Constraint or Directional Consistency Constraint, respectively, and 1.05 percentage points higher than standard WIoU. This result strongly demonstrates that the combination of the two constraints is not a simple linear addition, but rather produces a synergistic effect that jointly optimizes bounding box regression for irregular small targets, thereby significantly improving the model’s localization accuracy.

### 4.3. Visualization Analysis Experiments

To gain deeper insights into model behavior, validate the effectiveness of the proposed improvements, and reveal the underlying mechanisms for performance enhancement, we conducted a comprehensive visual analysis. These visualization results demonstrate the model’s detection capabilities, attention mechanisms, training stability, and data characteristics from multiple perspectives, providing compelling empirical evidence for understanding the model’s superior performance.

**Figure 6:**
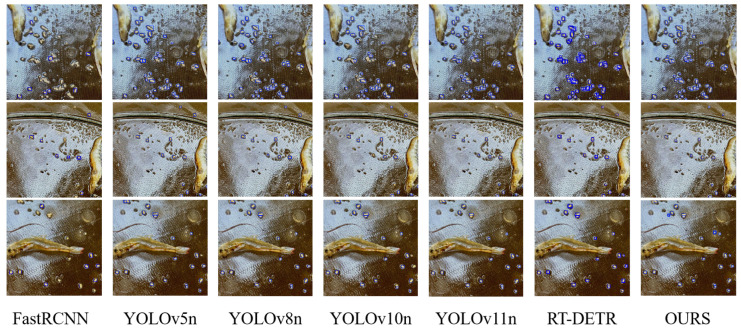
Detection Results of Different Algorithms in Various Typical Scenarios. The proposed YOLO-Shrimp model (last column) significantly reduces the missed detection rate in high-density areas compared to other methods, demonstrating its superior performance in complex, real-world conditions.

Figure 6 presents the detection results of our model across various representative scenarios. To comprehensively evaluate model robustness, we intentionally selected challenging cases including high-density and low-density residual feed distributions, varying illumination conditions, and complex background interference. Several notable characteristics can be observed from these detection results: First, in scenarios with extremely dense residual feed particles, the model accurately identifies most mutually adherent and overlapping particles, which benefits from the EnSimAM attention mechanism’s enhancement of local features and edge information. Second, for exceptionally small residual targets, the model maintains precise localization with bounding boxes closely aligned to actual targets, validating the optimization effect of the EnWIoU loss function for small object detection. Furthermore, under uneven illumination or turbid water conditions, the model exhibits only minor performance degradation, demonstrating excellent environmental adaptability. Notably, the model achieves remarkably high accuracy in determining the orientation of elongated residual particles, directly reflecting the effectiveness of the directional consistency constraint in EnWIoU. Overall, both the missed detection rate and false detection rate remain at minimal levels, providing visual evidence of our model’s robustness and practical utility.

**Figure 7:**
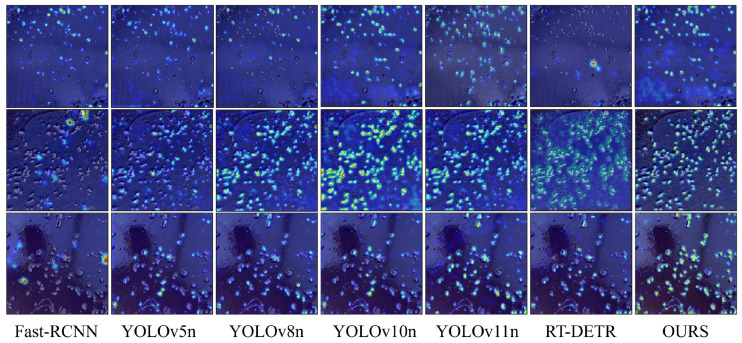
Heatmap Visualization (Red = High Activation; Blue = Low Activation). Compared to the baseline YOLOv11n, the heatmap for our model shows stronger and more focused activation at the boundaries of residual feed particles, indicating that the EnSimAM module effectively enhances feature representation for small targets.

To gain deeper insights into the operational principles of the YOLO-Shrimp mechanism, we employed Grad-CAM to generate model attention heatmaps as shown in Figure 7. Comparative analysis revealed several significant observations. The enhanced model exhibits more focused and precise activation patterns, with highlighted regions almost completely covering dense residual feed areas in the feeding trays while effectively suppressing activation in irrelevant regions such as tray edges, water surface reflections, and other background elements. This demonstrates that EnSimAM successfully guides the model to concentrate computational resources on task-relevant targets, effectively mitigating background interference. Comparison between baseline and improved models shows substantially enhanced activation intensity along small target edges in our approach, directly attributable to the edge response enhancement branch in EnSimAM. Furthermore, when processing dense small targets, the improved model achieves better distinction between adjacent objects and avoids excessive fusion of activation regions, benefiting from the local variance enhancement branch’s capacity for capturing fine-grained details. Finally, we observe complementary activation patterns across different feature scales: shallow layers focus more on detailed edges while deeper layers emphasize semantic information and global context, validating the effectiveness of our multi-scale attention fusion strategy.

**Figure 8:**
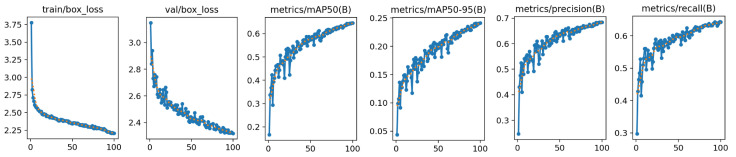
Detailed Indicator Changes of the Model During Training.

Figure 8 illustrates the detailed evolution of key training metrics, including total loss, bounding box regression loss, classification loss, and mAP. The training curves reveal that all loss functions exhibit smooth descending trends without significant oscillations or instability, demonstrating the numerical stability of our proposed EnWIoU loss function. Particularly during the initial training phase, the bounding box regression loss decreases more rapidly compared to the baseline model, which can be attributed to EnWIoU’s refined constraints on small target morphology and orientation, enabling faster acquisition of effective localization strategies. The mAP@0.5 metric shows consistent improvement on both training and validation sets, with the performance gap between them remaining minimal, indicating strong generalization capability without overfitting. Notably, the validation mAP continues to improve gradually during later training stages, suggesting the model avoids premature convergence to local optima. Furthermore, analysis of AP variations across different target sizes reveals that small targets achieve the most substantial improvement, directly validating the targeted optimization of our approach for small object detection.

**Figure 9:**
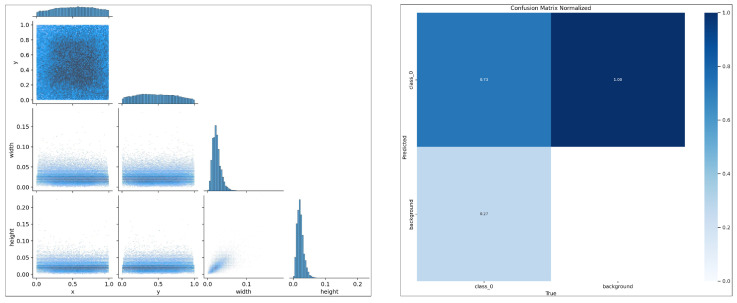
Detailed Content of the Confusion Matrix.

Figure 9 presents the statistical characteristics of the dataset and the predictive performance analysis of the model. Regarding data distribution, we conducted detailed statistics on target sizes, locations, and morphological features. The results indicate that approximately 85% of targets in the dataset fall into the small-size category, confirming the small-object nature of the residual feed detection task. The spatial distribution heatmap reveals that targets are primarily concentrated in the central image region, consistent with the actual positioning of feeding trays and demonstrating the standardization of our data collection process. In terms of morphological distribution, approximately 40% of targets exhibit elongated characteristics, which motivated the incorporation of aspect ratio constraints in our EnWIoU design. Confusion matrix analysis provides detailed insights into the model’s classification performance. The matrix demonstrates exceptionally high recognition accuracy for the residual feed category, with remarkably low false positive rates and false negative rates. These results indicate superior precision and minimal misclassification. Particularly noteworthy is that the majority of false negative cases arise from extremely small or severely blurred targets rather than systematic model errors, further validating the robustness of our approach.

### 4.4. Discussion on Generalization and Limitations

It is important to acknowledge that the data used for training and validating YOLO-Shrimp were collected from a single aquaculture farm. This presents a potential limitation regarding the model’s generalization capabilities when deployed in different environments. Specifically, the model may encounter a domain shift, where the statistical distribution of data in a new environment differs from the training data, potentially leading to a degradation in performance. Such shifts can be caused by a variety of factors, including differences in feeding tray materials, variations in camera setup, and diverse aquatic conditions. Therefore, while our current results are promising, future work should focus on evaluating and enhancing the model’s robustness across multiple, diverse aquaculture sites, as outlined in our future work section.

In addition to domain generalization, the model has other inherent limitations; while YOLO-Shrimp demonstrates strong performance, its detection accuracy may decline when faced with extremely minuscule or severely occluded residual particles, as critical feature information becomes indistinguishable from background noise. The model’s performance is also predicated on reasonable image quality; high water turbidity or poor illumination can obscure targets and degrade accuracy. Furthermore, a crucial trade-off exists between model complexity and performance; while YOLO-Shrimp is lightweight, deploying it on edge devices with significantly less computational power than our test setup would necessitate further model compression or a reduction in input resolution. Reducing the input resolution would accelerate inference but could render the smallest targets undetectable. Conversely, further pruning the model’s parameters might compromise its feature extraction capacity, which is vital for discerning subtle targets. This balance must be carefully calibrated based on the specific hardware constraints and the minimum required detection accuracy for a given application.

### 4.5. Practical Deployment Considerations

To bridge the gap between research and practical application, a typical deployment scheme for YOLO-Shrimp in a farm environment is envisioned as follows. A waterproof camera is installed at a fixed height directly above each feeding tray, equipped with a supplementary, consistent light source to mitigate variations in ambient lighting. Post-feeding, images are captured at a low frequency, such as once every 15 to 30 min. The image processing pipeline, running on an edge computing device like an NVIDIA Jetson Orin Nano, would execute the following steps: image acquisition, preprocessing, YOLO-Shrimp inference, and post-processing. The final output—a quantitative measure of residual feed—is then transmitted to a central farm management system. This data can trigger real-time alerts to the farm manager if residuals exceed a critical threshold or be integrated into an automated feeding control system to adjust the quantity for the next feeding cycle, thereby optimizing feed usage.

Regarding performance on representative edge hardware, while we did not conduct physical deployment tests, we can estimate the performance based on the model’s complexity. YOLO-Shrimp has a computational load of 5.5 GFLOPs. On a device like the NVIDIA Jetson Orin Nano, which is designed for such AI workloads, it is reasonable to expect a high throughput after optimization with tools like TensorRT. We estimate the end-to-end latency, including image capture and all processing steps, to be well under 500 ms, with an inference throughput potentially exceeding 50 frames per second. Given that feeding adjustments are made on a scale of hours, this near-real-time performance is more than sufficient to meet the operational requirements of a precision aquaculture feeding system, providing timely and actionable data for decision-making.

### 4.6. Failure Case Analysis

**Figure 10:**
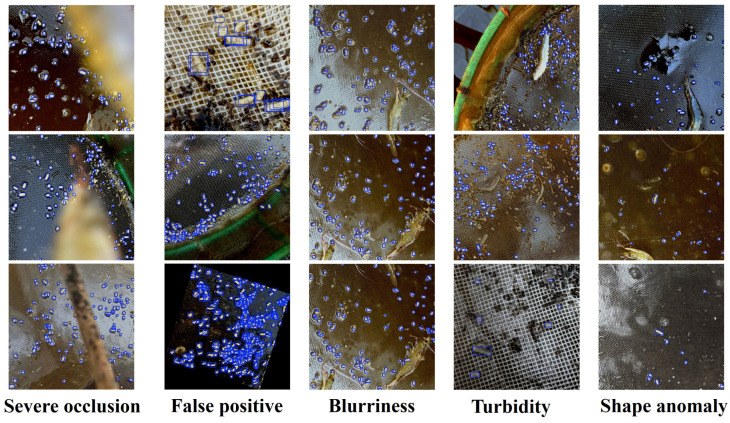
Visualization of YOLO-Shrimp Failure Cases in Challenging Scenarios. The examples illustrate that the model’s performance is most challenged by severe occlusion, visually similar distractors causing false positives, motion blur, high water turbidity, and particles with anomalous shapes.

To provide a balanced perspective on the model’s capabilities, we analyzed its performance under particularly challenging conditions. Figure 10 presents a gallery of representative failure cases, categorized by the primary cause of error. While YOLO-Shrimp is generally robust, its performance can be compromised in several specific scenarios. (1) Severe occlusion, where residual feed is hidden by shrimp or large pieces of debris, results in missed detections as key visual features are obscured. (2) False positives occasionally occur when non-feed items, such as small air bubbles, shrimp excrement, or reflective glints on the tray mesh, share visual characteristics with feed particles. (3) Blurriness, caused by rapid water movement or camera motion, degrades the fine-grained details necessary for accurate localization. (4) High turbidity reduces image contrast, causing feed particles to blend into the background and become difficult to distinguish. (5) Shape anomaly, where particles are broken, swollen, or clumped together, leads to detection failures because their morphology deviates significantly from the typical elongated shapes learned during training. These cases highlight the remaining challenges and suggest that future work could focus on improving model robustness against severe occlusion and developing more discriminative features to distinguish between feed and visually similar distractors.

## 5. Conclusions and Future Work

This paper addresses the critical need for automated residual feed detection in intensive shrimp farming by proposing YOLO-Shrimp, a lightweight yet high-precision object detection method based on YOLOv11n. To overcome the challenges posed by the small size, dense distribution, and morphological diversity of residual feed particles, we have implemented systematic optimizations across three key aspects: model architecture, attention mechanisms, and loss function design. Specifically, we introduced RepGhost modules to construct a hardware-efficient lightweight backbone network; developed EnSimAM, a multi-scale attention mechanism that integrates global, local, and edge information to enhance feature extraction for small targets; and proposed EnWIoU, an enhanced loss function that incorporates target morphology and orientation constraints to improve localization accuracy for small objects. Through rigorous experimental evaluation, we have comprehensively validated the effectiveness of our approach. Compared with the baseline YOLOv11n and other state-of-the-art detection models, our method achieves superior performance on our real-world dataset, attaining 70.01% mAP@0.5 while reducing parameter count and computational cost to 2.08M and 5.5 GFLOPs, respectively. Looking ahead, despite YOLO-Shrimp’s robust performance for its target task, several key directions remain for advancing its capabilities: we will first evolve the single-task framework into a multi-task system that simultaneously detects residual feed and analyzes shrimp feeding behaviors (e.g., aggregation density, activity levels), which enables a behavior-driven closed-loop feeding strategy via correlating feed consumption with behaviors by adopting a shared backbone with task-specific heads for joint training to leverage cross-task synergies. Furthermore, to enhance practical utility, we will extend the model to multi-class detection for distinguishing residual feed, shrimp feces, and other debris, which requires adapting the detection head from single- to multi-class prediction, updating the classification loss, and optimizing the EnWIoU loss into a class-aware adaptive version to accommodate diverse morphologies of new objects. To ensure broader applicability across diverse aquaculture environments (with variations in lighting, turbidity, tray designs, and shrimp species), we will also improve the model’s robustness using unsupervised/semi-supervised domain adaptation, allowing generalization from the labeled source dataset to unlabeled/sparsely labeled target farms to reduce re-annotation efforts and enhance scalability. In the long term, our goal is to develop a unified, flexible intelligent aquaculture monitoring framework via joint optimization of architectures and loss functions, exploring AutoML approaches to automatically discover optimal network-module and loss-component combinations for diverse tasks such as fish counting, disease detection, and growth assessment, thereby moving toward a comprehensive, adaptable intelligent monitoring platform for precision aquaculture.

## Figures and Tables

**Table 1:** Training and Inference Process of YOLO-Shrimp.

**Input** Image *I*, Ground-truth boxes $B_{\mathrm{gt}}$
**Output** Predicted boxes $B_{\mathrm{pred}}$
**Initialization**
Model=YOLO_Shrimp(backbone=’RepGhost’,attention=’EnSimAM’)
Optimizer=Adam(Model.parameters())
**Training Phase**
**for** epoch=1 **to** max_epochs **do**:
**for** $I,B_{\mathrm{gt}}$ **in** dataloader **do**:
Features_backbone=Model.backbone(I)
Features_neck=Model.neck(Features_backbone)
$P_{\mathrm{pred}},B_{\mathrm{pred}\text{\_}\mathrm{raw}}=\mathrm{Model}.\mathrm{head}\left(\mathrm{Features}\text{\_}\mathrm{neck}\right)$

$\mathrm{Loss}\text{\_}\mathrm{obj}=\mathrm{BCELoss}(P_{\mathrm{pred}},P_{\mathrm{gt}})$
$\mathrm{Loss}\text{\_}\mathrm{cls}=\mathrm{CE}\text{\_}\mathrm{Loss}(P_{\mathrm{pred}},P_{\mathrm{gt}})$
$\mathrm{Loss}\text{\_}\mathrm{loc}=\mathrm{EnWIoU}(B_{\mathrm{pred}\text{\_}\mathrm{raw}},B_{\mathrm{gt}})$
$\mathrm{Total}\text{\_}\mathrm{Loss}=\lambda_{1}\mathrm{Loss}\text{\_}\mathrm{obj}+\lambda_{2}\mathrm{Loss}\text{\_}\mathrm{cls}+\lambda_{3}\mathrm{Loss}\text{\_}\mathrm{loc}$
Optimizer.zero_grad()
Total_Loss.backward()
Optimizer.step()
**end for**
**end for**
**Inference Phase**
Model.eval()
withtorch.no_grad():
$\mathrm{Features}\text{\_}\mathrm{backbone}=\mathrm{Model}.\mathrm{backbone}(I_{\mathrm{test}})$
Features_neck=Model.neck(Features_backbone)
$B_{\mathrm{pred}}=\mathrm{Model}.\mathrm{head}\left(\mathrm{Features}\text{\_}\mathrm{neck}\right)$
$B_{\mathrm{pred}}=\mathrm{NMS}\left(B_{\mathrm{pred}}\right)$
**return** $B_{\mathrm{pred}}$

**Table 2:** Hardware and Software Configuration.

Hardware/Software	Configuration
GPU	NVIDIA RTX 4090 (24 GB) (Nvidia, Santa Clara, CA, USA)
CPU	Intel(R) Xeon(R) Gold 5418Y (10 Cores) (Intel, Santa Clara, CA, USA)
Memory	120 GB
Operating System	Ubuntu 22.04
Deep Learning Framework	PyTorch 1.13.1
Python Version	3.9

**Table 3:** Results of Comparative Experiments, Where Bold Values Represent the Optimal.

Model	mAP@0.5	mAP@0.95	P	R	Params (M)	GFLOPs
SSD [19]	6.52	1.41	18.50	23.30	6.13	**3.04**
Faster R-CNN [44]	16.68	4.46	30.24	24.53	18.93	41.80
RT-DETR [45]	42.40	13.10	47.40	51.05	19.00	54.09
YOLOv5n [46]	62.60	23.06	67.07	62.34	2.19	5.92
YOLOv8n [47]	64.57	24.13	68.37	64.22	2.69	6.94
YOLOv9n [25]	63.57	23.91	67.69	63.07	1.77	6.7
YOLOv10n [48]	60.93	22.72	63.98	60.55	2.70	8.39
MobileNet-SSD [49]	18.52	4.86	32.15	30.72	2.35	3.20
RTMDet-tiny [50]	61.85	22.30	66.20	61.50	2.80	4.00
YOLOv11n [26]	65.88	25.08	69.28	64.25	2.59	6.44
Ours	**70.01**	**28.01**	**72.53**	**65.88**	**2.08**	5.50

**Table 4:** Ablation Experiments (R = RepGhost, E = EnSimAM, U = EnWIoU).

R	E	U	mAP@0.5	mAP@0.95	P	R	Params (M)	GFLOPs
			65.88	25.08	69.28	64.25	2.59	6.44
✓			68.68	27.39	70.91	66.09	2.08	5.50
	✓		67.15	25.75	70.02	64.92	2.59	6.44
		✓	66.93	25.72	69.66	64.84	2.59	6.44
✓	✓	✓	70.01	28.01	72.53	65.88	2.08	5.50

**Table 5:** Performance Comparison of Different Augmentation Strategies for YOLO-Shrimp.

Model/Strategy	mAP@0.5	mAP@0.95	P	R	Params (M)	GFLOPs
No Augmentation	65.32	24.17	68.21	61.05	2.08	5.50
Full Data Augmentation	68.15	26.42	70.38	63.72	2.08	5.50
Local Data Augmentation	69.24	27.18	71.65	64.90	2.08	5.50
Full + Local Augmentation	70.01	28.01	72.53	65.88	2.08	5.50

**Table 6:** Fine-grained Ablation Experiments of EnSimAM and EnWIoU Modules.

Module	Component	mAP@0.5	mAP@0.5:0.95	ΔmAP@0.5
Baseline	–	65.88	25.08	–
EnSimAM	+ Global	66.58	25.41	+0.70
	+ Global + Local	66.88	25.63	+1.00
	+ Global + Local + Edge	67.15	25.75	+1.27
EnWIoU	+ WIoU (Baseline)	65.88	25.08	–
	+ WIoU + Aspect	66.30	25.33	+0.42
	+ WIoU + Direction	66.13	25.21	+0.25
	+ WIoU + Aspect + Direction	66.93	25.72	+1.05

## Data Availability

The data presented in this study are available upon request from the corresponding author. The data are not publicly available due to it being only available to teams interested in collaboration.
